# Life-threatening complications and intensive care unit management in patients treated with blinatumomab for B-cell acute lymphoblastic leukemia

**DOI:** 10.1186/s13054-023-04787-x

**Published:** 2024-01-05

**Authors:** Irene Urbino, Etienne Lengliné, Sandrine Valade, Marco Cerrano, Marie Sebert, Emmanuel Raffoux, Florence Rabian, Elie Azoulay, Nicolas Boissel

**Affiliations:** 1 Hématologie Clinique, Hôpital Saint-Louis, APHP, Université Paris Diderot, Paris, France; 2 Division of Hematology, Department of Oncology, AOU Città della Salute e della Scienza di Torino, Turin, Italy; 3 Medical Intensive Care Unit, Hôpital Saint-Louis, APHP, Université Paris Diderot, Paris, France

Blinatumomab, a CD3/CD19 bispecific antibody categorized under BiTEs (bispecific T-cell engagers), has recently gained approval as a standard of care in B-cell acute lymphoblastic leukemia (B-ALL) [1, 2]. Despite its efficacy, the emergence of specific toxicities, such as cytokine release syndrome (CRS) and immune effector cell-associated neurotoxicity syndrome (ICANS) with potential life-threatening grades, has been noted [3]. To date, few data are available in the literature on this emerging concern despite a very broad development of BiTEs in various diseases. This research letter aims to analyze life-threatening complications (LTCs), their rate, determinants and outcomes in a large cohort of patients treated with blinatumomab.

We conducted a retrospective analysis of 116 consecutive B-ALL patients treated with blinatumomab (cycles 2–5) outside clinical trials between April 2012 and June 2021 at Saint-Louis Hospital in Paris, France (*n* = 101), and AOU Città della Salute e della Scienza in Turin, Italy (*n* = 15). LTC was defined as grade ≥ 4 in common terminology criteria for adverse events V5 classification or the need for any life-sustaining therapy. All data were extracted from the electronic medical records. Adverse events were identified according to consensus recommendations by manual monitoring of the physician's diagnoses mentioned in the records and the study adhered to the Declaration of Helsinki.

Among 116 patients, 99 were treated while in complete remission.

CRS occurred in 59 (51%) (grade 1–2, 49%; grade ≥ 3, 2%) and neurotoxicity in 30 (26%) with 10% grade ≥ 3. Severe hematological toxicity (grade ≥ 3) was observed in 20 (17%) patients, and 46 (40%) had at least one infection during blinatumomab treatment.

Life-threatening complications were observed in 10 (9%) patients out of 321 blinatumomab cycles (Table 1). LTC occurred during the first cycle in 60% and while in complete remission in 80%. Eight patients required an intensive care unit (ICU) admission. Primary reason for admission was sepsis in 3 patients, ICANS in 3 patients and CRS in 2 patients. Median sequential organ failure assessment (SOFA) was 3.5, and 62% were hypotensive. In all blinatumomab was stopped, 3 received dexamethasone, none received tocilizumab. Five among ICU admitted patients had an infection, mostly bacterial bloodstream ones. Median time of onset in the three patients with ICANS was of 10 days. Two had status epilepticus and one non-epileptic motor weakness and consciousness alteration. All required invasive protective mechanical ventilation (24-72 h). Full recovery was observed in all cases after dexamethasone and anti-epileptic drugs, and blinatumomab could be resumed in 2 patients with prophylaxis. Both patients with CRS were admitted to ICU because of grade 2 hypotension occurring within 48 h from the start of blinatumomab infusion. Both had favorable evolution with fluid expansion and blinatumomab interruption without vasopressor, and blinatumomab could be resumed subsequently.

**Table 1:** Characteristics of life-threatening complications observed in blinatumomab-treated patients

	No. of patients (%)
Patients with life-threatening event	10
Blinatumomab cycle	
1st cycle	6 (60)
2nd cycle	2 (20)
3rd or > cycle	2 (20)
Inpatient monitoring for first days’ administration	10 (100)
Pre-blinatumomab status	
CR MRD neg	4 (40)
CR MRD pos	4 (40)
Not CR	2 (20)
Blinatumomab schedule	
9—28 mcg/day	4 (40)
28 mcg/day	6 (60)
Toxicity cause	
CRS	3 (30)
ICANS	3 (30)
CRS and ICANS	1 (10)
Clinical sepsis	3 (30)
Documented infection	5 (50)
Clinical	1
Microbiological	4
Characteristics at ICU admission (*n* = 8)	
Fever, *n*° (%)	4 (50)
Hypotension, *n*° (%)	5 (62.5)
Hypoxemia, *n*° (%)	0 (0)
SOFA score, median (range)	3.5 (1–6)
KDIGO, median (range)	0 (0–0)
GCS, median (range)	15 (6–15)
Interventions	
Dexamethasone	4 (40)
Tocilizumab	0 (0)
Antibiotic therapy	9 (90)
Fluid replacement	6 (60)
Vasoactive drugs	2 (20)
Mechanical ventilation	3 (30)
Renal replacement therapy	0 (0)
Deaths	3 (30)
Death cause	
CRS only	1
CRS and ICANS	1
Infection	1

CR: complete remission, MRD: measurable residual disease, CRS: cytokine release syndrome, ICANS: immune effector cell-associated neurotoxicity syndrome, ICU: intensive care unit, SOFA: sequential organ failure assessment, KDIGO: kidney disease improving global outcomes, GCS: glasgow coma scale

No specific patient or disease (age, sex, performance status, measurable residual disease level, white blood cell count, neutrophils and lymphocytes count, nervous system infiltration, previous allogenous stem cell transplantation, c-reactive protein level) characteristics were significantly associated with LTC occurrence. Three patients experienced death possibly related to blinatumomab according to the clinician in charge, all aged over 50 years. Of them, two patients were not admitted to the ICU given the underlying ALL poor prognosis. Broad-spectrum antibiotics were initiated for patients with LTC, regardless of specific signs of infection.

Life-threatening events occurred in 9% of B-ALL patients treated with blinatumomab in our cohort, necessitating ICU admission in 7% of the cases, which compares favorably with the rate observed after intensive chemotherapy (17.5%) [4] or CAR-T cells (32%) [5]. A low tumor burden before treatment did not seem to be protective against the occurrence of neurological and/or infectious LTC and, given the lack of determinants associated with LTC occurrence, a close monitoring in both inpatient and outpatient settings remains crucial. Infection emerged as the most common serious complication; thus, broad-spectrum antibiotic therapy initiation is reasonable in blinatumomab-treated patients experiencing LTC. Intensivist should be aware of these specific complications and the possible high rate of reversibility may help triage decisions in difficult cases. Although to our knowledge this is the only study describing post-blinatumomab LTC in the literature, the relevance of our results is limited by its retrospective and bicentric nature. Therefore, further studies focused on complications of emerging T-cell engager's drugs could certainly be useful.

With several bispecific antibodies poised to enter clinical practice, shared knowledge of potential severe complications is important for future clinical management. Our findings contribute valuable insights into the complexities associated with blinatumomab treatment, emphasizing the need for continued research to optimize patient outcomes.

## Data Availability

The datasets used and/or analyzed in the current study are available from the corresponding author on reasonable request.

## Abbreviations

B-ALL: B-cell acute lymphoblastic leukemia
BiTE: Bispecific T-cell engager
CAR: Chimeric antigen receptor
CRS: Cytokine release syndrome
ICANS: Immune effector cell-associated neurotoxicity syndrome
ICU: Intensive care unit
LTC: Life-threatening complications
SOFA: Sequential organ failure assessment
